# The Impact of Competitive Fatigue on Physiological Variables in National Level Youth Swimmers

**DOI:** 10.3390/jfmk10030256

**Published:** 2025-07-05

**Authors:** Alejandro López-Hernández, Anthony P. Turner, Hui Kwan Nicholas Lam, Juan Angel Simón-Piqueras, Violeta Muñoz de la Cruz, José María González Ravé

**Affiliations:** 1 Sports Training Laboratory, Faculty of Sports Sciences, University of Castilla La Mancha, Carlos III Avenue, 45071 Toledo, Spain; violeta.munoz@uclm.es; 2 Sport, Physical Education & Health Sciences, University of Edinburgh, Edinburgh EH8 8AQ, UK; tony.turner@ed.ac.uk (A.P.T.); nicholas.lam@ed.ac.uk (H.K.N.L.); 3 Faculty of Education, University of Castilla La Mancha, Albacete Campus, 02001 Albacete, Spain; juanangel.simon@uclm.es

**Keywords:** youth swimmers, blood lactate, heart rate, CMJ, RPE, swimming performance, physiological monitoring, neuromuscular fatigue

## Abstract

**Background:** This study investigated the impact of competitive fatigue on physiological (blood lactate and heart rate [HR]), neuromuscular (countermovement jump [CMJ]), and psychological (rate of perceived exertion [RPE]) variables in youth swimmers. **Methods:** Forty-two swimmers (mean ± SD, 14 ± 0.5 years; height for boys: 1.73 ± 0.03 m, girls: 1.69 ± 0.02 m; body mass: 67 ± 2.8 kg for boys and 62 ± 2.8 kg for girls) participated during a four-day championship, with data collected before and after their competition heats. **Results:** Significant post-race increases in lactate levels (*p* < 0.05) and RPE (*p* < 0.05) were observed across all distances, particularly after the 100 m and 200+ m events. Heart rate showed a decrease after the 50 m event and an increase after longer distances, reflecting different recovery demands. Although CMJ performance decreased after the races, these changes were not statistically significant (*p* = 0.810). The findings underscore the importance of race distance in modulating fatigue responses and suggest that RPE and lactate are the most sensitive indicators of competition-induced stress in these youth swimmers. **Conclusions:** Lactate accumulation and perceived exertion were the most sensitive indicators of fatigue (both *p* < 0.01), while HR and CMJ responses exhibited variability depending on race distance. These findings highlight the practical use of lactate and RPE monitoring related to fatigue and recovery strategies during competitions in youth swimmers.

## 1. Introduction

In swimming competitions, athletes often exert maximum performance with little time to rest between events, increasing the possibility of lactate accumulation [1] and affecting other key variables such as the rating of perceived exertion (RPE) [2] or countermovement jump (CMJ) [3,4]. The heavy demands of training for competitive swimming might cause local muscular fatigue and inhibit the development of maximal swimming power during periods of the training cycle, although a well-structured plan could lead to significant age-group swimming performance improvements [4,5,6]. Several studies focused on analyzing physiological variables of fatigue, both in training and in competition, including aspects such as warming up [7]. Others have explored the relationship between training and fatigue through variables such as heart rate variability (HRV) [8], providing insights into determining the optimal physiological state for swimmers to compete. A recent article by Sorgente [9] focused on determining fundamental recovery protocols for the optimal development of swimmers. These protocols are of vital importance for ensuring the optimal performance of swimmers at various specific moments of the championship, where they must perform at their highest level to ensure progressive qualification, and then in the finals. The populations studied in their research are similar to those in the present study.

Some relationships between physiological indicators relating to a swimmer’s performance, fatigue, and vertical jump (CMJ) have been established. For example, Keiner et al. [10] reported several strong correlations between a range of strength tests (bench press, squats, and countermovement jumps (CMJ)) and swimming performance for sprints between 15 and 100 m and, particularly, for the shorter distances up to 25 m.

International swimming championships typically span seven days during the European Championships, eight days for the World Championships, and nine days at the Olympic Games [11]. Competition formats in many national contexts replicate these demands over four days, with heats, finals, and relays [12]. For swimmers aged between 13 and 14 years, international championships (such as the European Youth Olympic Festival (EYOF) or Mediterranean Swimming-COMEN cup) usually last around three to five days, featuring morning heats and afternoon finals along with relay events. Training and competitive swimming for young athletes already adopt formats similar to those of international adult swimming, exposing them to periods of stress repeatedly within a short timeframe. Exposure to stressors such as unfamiliar environments, new competitors, and high-stakes performances increases the likelihood of both psychological and physiological fatigue. However, limited research has analyzed real-time fatigue responses in adolescent swimmers under live competition settings. Recent studies, like those by Zacca et al. [5] and Ferreira et al. [4,6], emphasize the importance of monitoring fatigue through multiple variables across training and competition cycles.

However, there is a lack of studies examining how race-induced fatigue may be modulated in less experienced athletes participating in such events, where they are exposed to multiple uncontrolled variables—including unfamiliar environments, novel competitors, altered daily routines, non-standard accommodations, and both external and internal performance pressures originating from coaches, family members, or the athletes themselves. Consequently, swimmers’ perceptions of fatigue and physiological exhaustion may be amplified by a range of psychological and physiological stressors, many of which remain difficult to monitor or regulate with the tools typically available to coaches and athletes [1,13]. Such research has the potential to contribute to the limited body of knowledge in this field by providing guidelines on how to navigate this type of competition. It would offer coaches tools to develop optimal competition strategies by considering physiological and psychological fatigue variables.

Accordingly, this study investigated the physiological (blood lactate and HR), neuromuscular (CMJ), and psychological (RPE) responses of national-level youth swimmers aged 13–14 years following sprint and middle-distance events in a competitive setting. We hypothesized that competitive swimming events elicit measurable physiological (blood lactate and heart rate), neuromuscular (countermovement jump), and psychological (rate of perceived exertion) responses in national-level youth swimmers aged 13–14 years.

## 2. Materials and Methods

### 2.1. Participants

The participants in this study comprised forty-two (30 males and 12 females) national swimmers (mean ± SD, 14 ± 0.5 years; height for boys: 1.73 ± 0.03 m, girls: 1.69 ± 0.02 m; body mass: 67 ± 2.8 kg for boys and 62 ± 2.8 kg for girls). All participants had 7.5 ± 1.2 years of swimming experience and achieved an average of 647 ± 22 FINA best performance points (World Aquatics, formerly FINA). We did not stratify by sex due to the limited sample size per subgroup. These swimmers were trained by different coaches from different parts of the country where data were collected. All participants qualified for the national Championships, meeting the minimum standards set by the National Swimming Federation for different events and distances.

All swimmers were classified as Tier 3 (Tier 3: Highly Trained/National Level) according to the classification framework of McKay et al. [14]. This study was approved by the local ethics committee (CEIS-739611-H7M1) in a session held on 25 February 2024. All participants were minors, and written informed consent was obtained from both the participants and their legal guardians/parents, as required by the ethics committee. This study adhered to the principles of the Helsinki Declaration.

To minimize potential disruptions to finals swimming performances, we only collected data during the heats in which they competed in the National Swimming Championship (50, 100, 200, and 400 m). Data were collected from swimmers depending on their competition schedule (12 swimmers participated in 100 m events, 9 participated in 200 m events, 19 participated in 50 m events, and 2 participated in 400 m events).

### 2.2. Experimental Design

The research design was an observational study embedded in real competition settings and was planned specifically for the current study. Data were collected before and after each heat to capture changes in neuromuscular performance (CMJ), physiology (lactate, HR), as well as RPE. Data collection was conducted in an ecological setting under time-constrained conditions, both pre- and post-competition, due to the logistical demands and competitive priorities of the event.

All swimmers were familiar with all testing protocols since they were swimmers who were enrolled in a talent identification program run by each regional governing body affiliated with the national swimming federation. Two experienced investigators performed all tests and visually verified them to ensure reproducibility of the technique and application of the protocol. The intraclass correlation coefficients (ICC) for CMJ pre/post for reliability were 0.87 (preparatory period) and 0.89 (competition period), indicating good test–retest consistency.

CMJ performance was assessed using the Optojump-next system (Microgate, Bolzano, Italy), which has been validated for measuring vertical jump height with high reliability in both laboratory and field conditions [15]. Swimmers performed two attempts before the race and two attempts after, with the highest value from each time-point being used for analysis. Capillary blood lactate (mmol/L) was withdrawn employing the finger-stick method (middle or ring finger) and was measured using the Lactate Scout analyzer (SensLab GmbH, Leipzig, Germany), a validated method for use in swimmers under competitive conditions [16]. Heart rate (HR) was recorded using a Polar Verity Sense optical sensor (Polar Electro Oy, Kempele, Finland) placed on the swimmer’s temple. The device captured a 5 s average immediately upon post-race application, minimizing interference and capturing acute cardiovascular response. This method has been shown to be suitable for field-based athlete monitoring [17].

The RPE was assessed using the category-ratio scale from 0 to 10 (CR10) developed by Borg [18]. RPE data were collected exactly five minutes after each race, before any recovery activity or feedback from coaches, and in a quiet area near the recovery zone to minimize external influence. Participants were asked to rate their perceived exertion during the competition event they had just completed. All swimmers were previously familiarized with the Borg CR10 scale as part of their regular training monitoring, reducing the risk of misinterpretation or response bias. A trained researcher administered the scale individually to each athlete to ensure consistency.

### 2.3. Procedure

The data collection was scheduled in the morning session (heats), a day of preliminary tests, where the swimmers seek classification for the afternoon finals. Due to the competition format, some swimmers swam several times during the four days of competition; therefore, some had more than one event in the morning and, in the case of qualifying, more than one final in the afternoon.

The first evaluation occurred just before the tests, called the pre-competition phase. The second evaluation occurred immediately after having completed the competition event and was called the post-competition phase. On all occasions, pre-competition data collection was performed 15 min before the start of the race, and post-competition data collection was performed 5 min after leaving the pool (having made maximum effort) and before performing any recovery protocol. The individual competition races were carried out in a 50 m indoor pool that had ten lanes, each 2 m wide.

**Figure 1:**
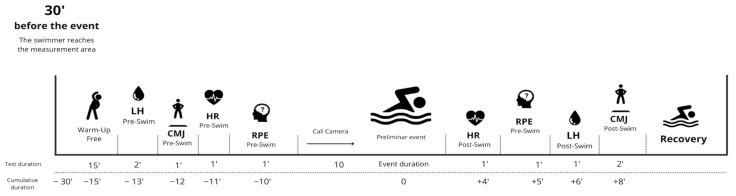
Overview of the experimental design, indicating the protocol and timing of measurements. LH, blood lactate; CMJ, countermovement jump; HR, heart rate; RPE, rating of perceived exertion.

These same experienced researchers (certified in sports performance assessment) were the ones who took the different physiological fatigue samples (HR, lactate, and RPE). All these data collections were carried out just before and after the races took place. The times of the swimming races were collected by professional staff of the Royal Spanish Swimming Federation using electronic touch panels (Ares 21 System). Data collection was carried out in a space enabled for this purpose, very close to the exit chamber and recovery area (Figure 1).

### 2.4. Statistical Analysis

All analyses were performed using R Statistical Software (RStudio, version 4.4.2) with the lme4 (v 1.1-37), lmerTest (v3.1-3), emmeans (v 1.11.1), and RLRsim (v3.1-8) packages. To examine changes in CMJ, lactate, HR, and RPE before and after swimming competitions, we first assessed whether incorporating random effects was necessary. This was performed using a restricted likelihood ratio test (RLRT), which compares a fixed-effects model to the model with random intercepts. A significant RLRT result indicated that a linear mixed-effects model (LMM) with a random intercept for the participants was more appropriate to account for within-subject variability; otherwise, a fixed-effects model was used. For all models, time (PRE vs. POST), race distance (50 m, 100 m, 200+ m), and their interaction were included as fixed effects. Race distance was modeled as a categorical variable with three levels: 50 m, 100 m, and 200+ m. In LMMs, we additionally tested whether including random slopes for time and distance improved model fit. Model selection was evaluated by comparing Akaike information criterion (AIC), Bayesian information criterion (BIC), and likelihood ratio tests. The model comparisons for CMJ indicated that adding random slopes for time or distance did not improve model fit compared to a random intercept-only model. In contrast, the random intercept plus a random slope for the time variable (PRE vs. POST) significantly improved model fit compared to other models and, therefore, was selected for further analysis of lactate. The final LMM was chosen based on the best fit (i.e., lowest AIC/BIC and significant model improvement). In LMM, fixed effects were evaluated using Satterthwaite-approximated degrees of freedom. For fixed-effects models, traditional F-tests and *t*-tests were used. Where significant main effects or interactions were found, post hoc pairwise comparisons were performed using estimated marginal means (EMMs) via the emmeans package (v 1.11.1). Tukey adjustments were applied for comparisons involving three or more levels (e.g., distance), whereas no adjustment was used for binary comparisons (e.g., PRE vs. POST). All data were presented as mean ± SD unless otherwise specified. Alpha was set at 0.05.

## 3. Results

**Figure 2:**
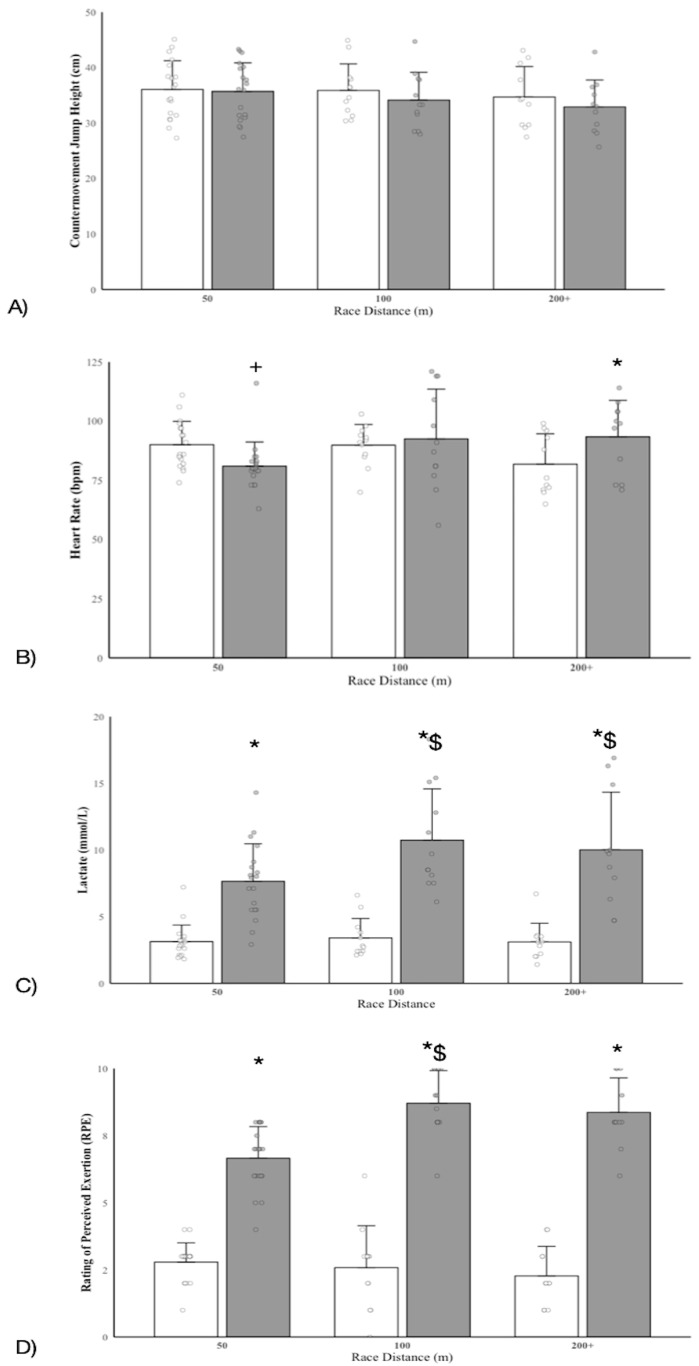
Changes in countermovement jump height (CMJ in cm, panel (**A**)), heart rate (HR in bpm, panel (**B**)), lactate (in mmol/L, panel (**C**)), and rating of perceived exertion (RPE in arbitrary units, panel (**D**)) before and after competition across race distances (50 m, 100 m, and 200+ m). White bars represent the overall mean at pre-competition, and grey bars represent the overall mean at post-competition. Dots represent individual participant scores, and error bars show SD. Mean ± SD. * significantly greater than equivalent pre-race value; + significantly lower than equivalent pre-race value; $ pre–post changes significantly larger than for 50 m race.

The overall changes for all outcome variables before and after swimming are presented in Figure 2.

An RLRT indicated that a random intercept for participant ID significantly improved model fit for CMJ (RLRT = 96.49, *p* < 0.001) and lactate (RLRT = 5.63, *p* = 0.007) but not for HR (RLRT = 1.26, *p* = 0.111) or RPE (RLRT = 0.73, *p* = 0.178). Therefore, a linear mixed-effects model was used for CMJ and lactate, while fixed-effects linear models were used for the other outcome variables.

CMJ: The LMM analysis revealed no significant main effect of time (β = −0.37, SE = 0.60, t(58.2) = −0.62, *p* = 0.539) and distance (100 m: β = −0.32, SE = 0.76, t(60.1) = −0.42, *p* = 0.697; 200+ m: β = −0.31, SE = 0.8, t(60.1) = −0.38, *p* = 0.704) and no significant interaction effects between time and distance (100 m: β = 1.38, SE = 0.97, t(58.2) = −1.42, *p* = 0.162; 200+ m: β = −1.41, SE = 1, t(58.2) = −1.41, *p* = 0.164). The model estimated a between-participant variance (σ^2^ = 22.17, SD = 4.71 cm) and a within-participant variance (σ^2^ = 3.48, SD = 1.86 cm), reflecting noticeable variability in baseline jump performance across participants (Table 1). There was a small decrease in CMJ height from PRE to POST in 50 m (d = −0.20, 95% CI [−0.85, 0.45]), 100 m (d = −0.47, 95% CI [−1.16, 0.22]), and 200+ m (d = −0.50, 95% CI [−1.2, 0.2]).

Lactate: The LMM with random intercepts for the participants was used because a more complex model including random slopes for time failed to converge (Table 1). Therefore, the model with the random intercept for the participant was retained. The LMM revealed a significant main effect of time (β = 4.53, SE = 0.77, t(59.1) = 5.86, *p* < 0.001), indicating higher lactate levels at POST compared to PRE (d = 1.51, 95% CI [0.92, 2.1]). No significant main effects were found for the 100 m (β = −0.05, SE = 0.91, t(70) = −0.05, *p* = 0.96, d = 0.01, 95% CI [−0.47, 0.46]) or 200+ m (β = 0.09, SE = 0.96, t(72.5) = 0.09, *p* = 0.93, d = 0.02, 95% CI [−0.47, 0.51]) races when compared to the 50 m race. There was a significant time x distance interaction for the 100 m race (β = 2.81, SE = 1.24, t(59.6) = 2.26, *p* = 0.027, d = 0.58, 95% CI [0.07, 1.09]), but it was not significant for the 200+ m race (β = 2.39, SE = 1.28, t(59.6) = 1.87, *p* = 0.067, d = 0.48, 95% CI [−0.03, 0.98]). Post hoc pairwise comparisons (with Satterthwaite-adjusted degrees of freedom) reported significant increases in lactate from PRE to POST across all distances: 50 m (t(59.1) = −5.86, *p* < 0.001), d = 1.51, 95% CI [0.92, 2.10]; 100 m (t(59.1) = −7.55, *p* < 0.001), d = 1.89, 95% CI [1.25, 2.52]); and 200+ m (t(59.1) = −6.81, *p* < 0.001), d = 1.73, 95% CI [1.11, 2.36]). Estimated marginal means showed that lactate increased from M = 3.17 mmol/L (SE = 0.48) at PRE to M = 9.44 mmol/L (SE = 0.48) at POST. At PRE, there were no significant differences in lactate between race distances (all *p* > 0.05). At POST-race, lactate concentrations were significantly higher for 100 m (M = 10.09, SE = 0.85) and 200+ m (M = 10.49, SE = 0.90) than for 50 m (M = 7.69, SE = 0.76), but Tukey post hoc comparisons were not statistically significant after adjustment (50 m vs. 100 m: *p* = 0.17; 50 m vs. 200+ m: *p* = 0.12; 100 m vs. 200+ m: *p* = 0.87). Random effects estimates indicated substantial variability in baseline lactate response, with a random intercept variance of σ^2^ = 7.68, SD = 2.77 mmol/L. The residual variance was σ^2^ = 2.95, SD = 1.72 mmol/L, reflecting within-subject variability.

HR: A fixed-effects model was used to analyze HR. The overall model was statistically significant (F(5, 78) = 2.37, *p* = 0.047), with an R^2^ of 0.13 and an adjusted R^2^ of 0.08. There was a significant moderate decrease in HR from PRE to POST (β = −9.05, SE = 4.24, t(78) = −2.14, *p* = 0.036, d = −0.81, 95% CI [−1.55, −0.07]) in the 50 m race. There was also a significant time × distance interaction in the 200+ m race (β = 20.6, SE = 7.0, t(78) = 2.94, *p* = 0.004), with a moderate increase in HR from PRE to POST (d = 0.79, 95% CI [0.05, 1.53]). No significant change was reported for the 100 m race (*p* > 0.05). Post hoc analyses revealed no significant differences in HR during the PRE-race phase between the three different race categories, but the 50 m race had a significantly lower HR than the 200+ m race (*p* = 0.038) during the POST-race phase. However, no significant difference was found between the 50 m and 100 m races (*p* = 0.50) or between the 100 m and 200+ m races (*p* = 0.99).

RPE: The fixed-effects model revealed a significant effect of time and distance on RPE (F(5, 78) = 86.01, *p* < 0.001, R^2^ = 0.85). Post hoc tests showed that RPE increased significantly from PRE to POST at all distances: 50 m (M: 2.79 to 6.66, *p* < 0.001, d = 2.72, 95% CI [2.14, 3.30]), 100 m (M: 2.58 to 8.71, *p* < 0.001, d = 5.55, 95% CI [4.97, 6.13]), and 200 m+ (M: 2.27 to 8.36, *p* < 0.001, d = 4.29, 95% CI [3.72, 4.87]). Post-race RPE was significantly higher following the 100 m and 200 m+ races compared to the 50 m race (*p* < 0.001), with no significant difference between the 100 m and 200 m+ races (*p* = 0.076).

## 4. Discussion

This study demonstrates that physiological and perceptual fatigue markers—specifically lactate and RPE—are responsive to race-induced stress in youth swimmers. The most pronounced effects occurred in middle-distance events. While HR responses varied by distance, CMJ was not sensitive to change in this competition context. These findings align with previous work on energy system contributions in swimming [19] and fatigue responses in youth athletes [4,6]. Lactate concentration was consistently higher post-race across all events, with large effect sizes and meaningful changes, with longer distances (200–400 m) showing the greatest increase (69.20%), followed closely by the 100 m event (68.40%). The 50 m event exhibited a comparatively lower increase (57.67%). These results align with previous research indicating that shorter sprint events rely more on phosphagen energy systems, whereas longer events demand greater anaerobic glycolysis [19,20]. Consequently, the elevated lactate levels in middle-distance events highlight the increased metabolic demands [20]. Ribeiro et al. [21] support this by demonstrating that swimmers competing at extreme intensities exhibit distinct biomechanical and energetic profiles, with performance level influencing the extent of lactate accumulation and coordination adjustments. Moreover, Massini et al. [22] found that the accumulated oxygen deficit—a marker of anaerobic energy contribution—was significantly higher in middle-distance events compared to sprints, particularly among high-performing youth athletes, underscoring the metabolic complexity and anaerobic load involved in 200–400 m races. These findings corroborate the notion that, despite being classified as sprint events, middle-distance races in swimming impose substantial anaerobic demands that are reflected in the elevated post-race lactate concentrations.

Heart rate (HR) responses exhibited an event-dependent pattern. The 50 m event demonstrated a post-race HR decrease (−12.22%), possibly due to rapid recovery mechanisms or active recovery effects. In contrast, the 100 m and 200–400 m events showed HR increases (2.88% and 12.37%, respectively), reflecting sustained cardiovascular demand. While our findings partially align with previous studies on elite swimmers [23], the variability in HR responses suggests that individual adaptations and external factors (e.g., pre-race stress, warm-up intensity) may influence post-race HR values [24]. Heart rate responses during competition are subject to several confounding influences that may obscure underlying physiological trends. Factors such as anticipatory HR elevation due to pre-race anxiety, variability in individual warm-up intensity, and the psychological stress associated with high-stakes performance can all affect post-race HR readings [25]. These sources of variation are particularly relevant in youth athletes, who often display heightened emotional reactivity [26].

In field-based studies with young swimmers, HR measurement is commonly used as a non-invasive and accessible indicator of physiological strain. Devices such as optical heart rate sensors and chest strap monitors provide real-time feedback that helps coaches adjust training intensity accordingly. However, one of the key challenges in measuring HR during competition is accounting for external stressors that may elevate HR even before the race begins. Factors such as race anxiety, insufficient recovery from previous events, and inconsistent warm-up routines can all contribute to individual variability in HR readings. Thus, field-based HR assessments must be interpreted with consideration of psychological and environmental factors that could influence cardiovascular responses, as evidenced in the low variance in the fixed-effects model (R^2^ = 0.13).

Similarly, lactate measurements in competition settings are particularly useful for assessing the anaerobic contribution to energy metabolism [21,22]. However, obtaining capillary blood samples immediately after race completion in a controlled manner poses logistical challenges, especially when working with young athletes. The time-sensitive nature of lactate sampling means that even minor delays in blood collection can lead to fluctuations in recorded values, potentially impacting the accuracy of fatigue assessment. Standardizing post-race measurement protocols, including designated testing areas and trained personnel, can help improve the reliability of lactate data collected during live competitions.

CMJ performance showed no significant differences (Figure 2) in the main effects of time (*p* = 0.810) or distance (*p* = 0.447). The 50 m event showed a minor reduction (−0.84%), while the 100 m (−5.12%) and 200–400 m events (−5.47%) exhibited greater decreases. The lack of significant changes suggests that short-term competition-induced fatigue may not be sufficient to produce marked declines in CMJ performance in this population and context. Indeed, the much larger between-subject (SD = 4.69 cm) vs. within-subject (SD = 1.86 cm) variability in CMJ may underpin the lack of significant differences across distances at least, but it may yet be useful for within-participant comparisons. Furthermore, variations in training background (e.g., between swimmers and non-athletic swimmers regarding postural control and neuromuscular activation [27]), sex, and primary swimming stroke may contribute to individual differences in neuromuscular fatigue.

CMJ testing has gained popularity in competitive swimming due to its ability to indirectly assess lower-limb power, which is essential for starts and turns. Also, Carvalho et al. [4] reported that dry-land strength testing distinguishes elite from non-elite swimmers, confirming that sprint swimming performance levels can be differentiated by dry-land strength testing. In field applications with young swimmers, CMJ assessments are often conducted before and after training sessions to monitor fatigue accumulation over time [3]. However, performing CMJ tests in a competition setting presents additional challenges, including time constraints and the need for standardized jump execution. While optical measurement systems such as Optojump provide precise jump height data, ensuring consistency in test administration is critical for obtaining reliable results [15]. Additionally, the psychological state of the athlete may influence CMJ performance, as pre-race nervousness or post-race exhaustion can affect jump execution. In this study, we could find no evidence of any association between individual pre–post changes in CMJ height and changes in blood lactate, HR, or RPE when further exploring the data. Therefore, while CMJ remains a valuable tool for assessing neuromuscular status, its interpretation should be contextualized within the broader competition environment for such athletes.

Among all measured variables, RPE exhibited the most substantial change and had the largest effect sizes, with significant pre-to-post-race (*p* < 0.001) increases across all events (*p* < 0.001). The largest increases were observed in the 100 m (70.34%) and 200–400 m (72.84%) events, while the 50 m event showed a slightly lower but still considerable increase (56.56%). These results reinforce the sensitivity of RPE as a measure of internal workload and fatigue in swimmers [23,28]. Although subjective, RPE remains a practical tool for monitoring training loads and competition stress, particularly in young athletes who may not have well-developed physiological feedback mechanisms.

In field research with young swimmers, RPE is widely used due to its simplicity and effectiveness in gauging perceived fatigue levels. Athletes are typically asked to provide RPE ratings immediately after their race, allowing for real-time assessment of exertion. Psycharakis et al. [29] not only confirmed the validity and reliability of RPE in elite swimmers (r = 0.82–0.85) but also described a familiarization procedure in which swimmers completed the incremental test before data collection. However, one of the main challenges with RPE measurements is their inherent subjectivity, as individual pain tolerance, motivation levels, and race expectations can all influence reported scores, although Toubekis et al. [13] also showed that estimation of the session-RPE training load may be helpful for taper planning of young swimmers. Additionally, younger athletes may struggle with consistently interpreting and using the RPE scale, leading to potential variability in reported values. To improve the accuracy of RPE assessments, researchers and coaches employ repeated measures throughout training cycles, helping athletes become more familiar with self-evaluating or anchoring their exertion levels.

Our findings offer practical implications for coaches aiming to optimize performance in young swimmers. Given the high lactate levels observed post-warm-up, reducing warm-up intensity in high-stress competitions may help mitigate premature lactate accumulation and improve race performance, ensuring suitable recovery methods to maximize recovery between events. Additionally, the observed HR patterns suggest that individualized recovery strategies may be necessary based on race distance. While CMJ did not show statistically significant changes, it may still serve as a useful assessment tool in competition settings, particularly in short-course pools where its reliability has been demonstrated [3,4].

The relatively specialized sample (national-level youth swimmers) limits the generalizability of our findings to broader populations. Additionally, the absence of a control group prevents direct comparisons of competition-induced fatigue versus normal training fatigue. The heterogeneity of the sample in terms of sex may influence the outcomes, and the gender imbalance (30 males, 12 females) could affect generalizability and also the distribution across race distances. Specifically, of the 19 swimmers in the 50 m events, 14 were male, and 5 were female; of the 12 swimmers in the 100 m events, 9 were male, and 3 were female; of the 9 swimmers in the 200 m, 6 were male, and 3 were female; and of the 2 swimmers in the 400 m, both were male. The preferred swimming stroke may have also influenced the variability in physiological and neuromuscular responses. Limitations also include the absence of data from finals, no standardization of warm-up protocols, and individual variability in effort. Additionally, psychological factors (e.g., race anxiety) may have influenced HR and RPE values. Future studies should include hormonal markers and longitudinal tracking across full competition cycles.

## 5. Conclusions

This study provides an analysis of race-induced fatigue in a cohort of young swimmers, highlighting the interplay between physiological, neuromuscular, and psychological responses during competition. Lactate accumulation and perceived exertion were sensitive indicators of fatigue post-race, while HR and CMJ responses exhibited event-dependent variability in this sample. These findings illustrate the potential to use RPE and blood lactate to explore the effects of warm-ups and recovery strategies on race-induced fatigue in young, competitive swimmers during real competitions, with further clarification needed on the utility of CMJ and HR for such purposes, at least using the approaches employed for the population studied here.

## Figures and Tables

**Table 1:** The model comparison between random intercept model and random intercept plus random slopes model for pre- and post-swimming competition.

Variable	Model	AIC	BIC	Log-Likelihood	χ^2^	df	*p*
CMJ	Random Intercept (Participant ID)	417.6	429.7	−203.8	-	-	-
	Random Slope: Participant × Time	420.7	437.7	−203.4	0.87	2	0.648
	Random Slope: Participant × Distance	421.4	438.4	−203.7	0	0	-
	Random Slopes: Participant × Time × Distance	426	450.3	−203	1.37	3	0.713
Lactate	Random Intercept (Participant ID)	411.1	423.2	−200.5	-	-	-
	Random Slope: Participant × Time	386.4	403.4	−186.2	28.68	2	**<0.001**
	Random Slope: Participant × Distance	414.4	431.4	−200.2	0	0	-
	Random Slopes: Participant × Time × Distance	391.6	415.9	−185.8	28.83	3	**<0.001**

AIC: Akaike information criterion; BIC: Bayesian information criterion; CMJ: countermovement jump; χ^2^: chi-square statistic; df: degrees of freedom; *p*: *p*-value. *p*-values in bold indicate statistically significant differences.

## Data Availability

Data are available on request.
